# *Auricularia heimuer* Ameliorates Oxidative Stress and Inflammation to Inhibit Atherosclerosis Development in ApoE^−/−^ Mice

**DOI:** 10.3390/nu17172799

**Published:** 2025-08-28

**Authors:** Jundi Zhao, Siyu Ma, Yifan Hu, Jing Ling, Zhuqian Wang, Jingyu Wang, Junliang Chen, Yongfeng Zhang

**Affiliations:** 1 Engineering Research Center of Chinese Ministry of Education for Edible and Medicinal Fungi, Jilin Agricultural University, Changchun 130118, China; zhaojundi@mails.jlau.edu.cn (J.Z.); masiyu@mails.jlau.edu.cn (S.M.); huyifan@mails.jlau.edu.cn (Y.H.); lingjing@mails.jlau.edu.cn (J.L.); 2 Jilin Academy of Agricultural Sciences, Changchun 130033, China; 3 Science and Research Center for Edible Fungi of Qingyuan County, Qingyuan 323800, China; 4 College of Plant Protection, Jilin Agricultural University, Changchun 130118, China; 5 Clinical Research Center for Advanced Diagnostics and Therapeutics, Zhongshan People’s Hospital, Zhongshan 528403, China; wzq20482159@outlook.com

**Keywords:** atherosclerosis, *Auricularia heimuer*, oxidative stress, inflammation, Nrf2/NF-κB signaling pathway

## Abstract

**Background:** Atherosclerosis is a chronic vascular disease triggered by lipid accumulation. *Auricularia heimuer* is rich in various bioactive compounds that have anti-inflammatory, antioxidant, and hypolipidemic properties. The specific beneficial effects of *A. heimuer* on atherosclerosis and its underlying mechanisms require further investigation. **Methods**: In this study, ApoE^−/−^ mice were utilized as models of atherosclerosis induced by a high-fat diet (HFD) to investigate the effects of *A. heimuer.* Analyses of gut microbiota and serum metabolomics were conducted to elucidate the potential mechanism. **Results:** In HFD-fed ApoE^−/−^ mice, *A. heimuer* significantly inhibited the increase in body weight, regulated lipid levels, and alleviated aortic lesions. *A. heimuer* also modulated the abundance of intestinal flora such as *Akkermansia* and *Ruminococcus* and altered the levels of serum metabolites, including 12(S)-hydroxy-5Z,8Z,10E,14Z-eicosatetraenoic acid (12(S)-HETE) and N-acetyl galactosamine 4-sulfate. Furthermore, *A. heimuer* alleviated oxidative stress and inflammatory responses, thereby mitigating atherosclerosis via the Nrf2/NF-κB signaling pathway. **Conclusions:** These findings suggest that *A. heimuer* may serve as a potential therapeutic strategy for atherosclerosis.

## 1. Introduction

Atherosclerosis (AS) is a chronic vascular condition characterized by arterial narrowing due to plaque accumulation, primarily consisting of lipids, inflammatory cells, and connective tissue [1]. As plaques accumulate, the blood vessel walls thicken, resulting in the narrowing of the lumen and a reduction in blood flow [2]. When a plaque ruptures, it exposes thrombogenic substances to the bloodstream, thereby initiating the formation of blood clots [2]. AS is a major underlying cause of cardiovascular diseases, which represent leading causes of mortality in China, accounting for nearly 50% of all deaths [3]. Currently, common treatments for AS include blood lipid control, antiplatelet therapy, and glycemic control; commonly used therapeutic drugs include statins, aspirin, metformin, etc. [4,5,6]. However, these medications can cause side effects such as abnormal liver function and gastrointestinal discomfort or damage [4]. Thus, there is an urgent need for more effective and safer strategies for treating AS.

The early events of atherosclerosis entail the infiltration of low-density lipoprotein (LDL) into the vascular wall, triggering an inflammatory response characterized by the infiltration of inflammatory cells into endothelial cells, lipid uptake, and the oxidation of LDL [7]. This oxidized LDL further exacerbates the development of fatty streaks and atherosclerotic plaques by instigating a cascade of pathophysiological events, encompassing endothelial dysfunction, foam cell formation, and enhanced monocyte chemotaxis [8]. In a hyperlipidemic state, increased production of reactive oxygen species (ROS) induces oxidative stress, which enhances the oxidation of subendothelial LDL, generates various lipid oxidation products, and subsequently impairs endothelial function [9]. Nuclear factor E2-related factor 2 (Nrf2), a major redox regulator of antioxidant response, modulates the expression of downstream enzymes such as heme oxygenase-1 (HO-1) and superoxide dismutase 1 (SOD-1) to reduce ROS level sand thereby mitigate oxidative stress in AS [10]. The deposition and oxidation of LDL within the vascular wall lead to the release of inflammatory mediators, such as IL-6, which, in turn, activates the nuclear factor kappa-light-chain-enhancer of activated B cells (NF-κB) signaling pathway, thereby initiating an inflammatory response [11]. This activation of NF-κB is a key driver of inflammation, as evidenced by the correlation between its activity and the release of inflammatory factors within atherosclerotic lesions [12]. Emerging evidence suggests that the intestinal microbiota is implicated in the pathogenesis of AS [13]. Dysbiosis of the gut microbiota alters the host’s metabolism, which, in turn, influences the progression of AS [13]. Notably, an elevated abundance of certain short-chain fatty acid-producing bacteria, such as *Roseburia*, *Faecalibacterium*, and *Bacteroides*, in ApoE^−/−^ mice fed a high-fat diet (HFD) can attenuate AS [14].

Historically, natural medicines have been widely used for the prevention and treatment of various diseases due to their remarkable efficacy and favorable safety profiles. As common and nutritionally rich dietary components, edible fungi have garnered substantial research attention. Increasing evidence highlights their multifunctional bioactivities; for example, *Morchella esculenta* has been shown to attenuate cholesterol synthesis by activating the AMP-activated protein kinase α (AMPKα) signaling pathway and suppressing sterol regulatory element-binding protein 2 (SREBP2) expression, thereby exerting a protective effect against AS progression [15]. Similarly, *Pleurotus abieticola* can diminish ROS levels and mitigate oxidative stress by enhancing Nrf2 expression, contributing to the alleviation of AS [16]. *Auricularia heimuer*, belonging to the Auriculariaceae family, is the third-largest cultivated edible fungus globally [17]. It is primarily cultivated in northern China, the world’s main producer, using methods such as wood log and substitute material cultivation. *A. heimuer* exhibits a range of biological activities, including cholesterol-lowering, hypolipidemic [18], antioxidant, and anti-inflammatory effects [19]. For instance, the phenolics from *A. heimuer* demonstrate antioxidant capacity of free radicals and inhibit lipid peroxidation [20]. Polysaccharides from *A. heimuer* lower blood glucose levels and improve kidney function in rat models [21]. However, the anti-atherosclerotic efficacy and underlying mechanisms of *A. heimuer* have not been fully elucidated.

In this study, we hypothesized that *A. heimuer* could alleviate atherosclerosis by modulating inflammatory responses and oxidative stress. To test this, we established a high-fat diet (HFD)-induced atherosclerosis model in ApoE^−/−^ mice. Our comprehensive analysis of the intestinal microbiota and serum metabolome revealed that *A. heimuer* treatment significantly altered the gut microbial composition and metabolite profiles. These changes contributed to improved lipid metabolism, reduced aortic lesions, and the amelioration of atherosclerosis symptoms. Mechanistically, we demonstrated that these beneficial effects were achieved by regulating the Nrf2/NF-κB signaling pathway, thereby suppressing inflammation and oxidative stress. These findings provide a theoretical basis and empirical evidence supporting *A. heimuer* as a promising therapeutic strategy for atherosclerosis.

## 2. Materials and Methods

### 2.1. Animals and Treatment

**Figure 1:**
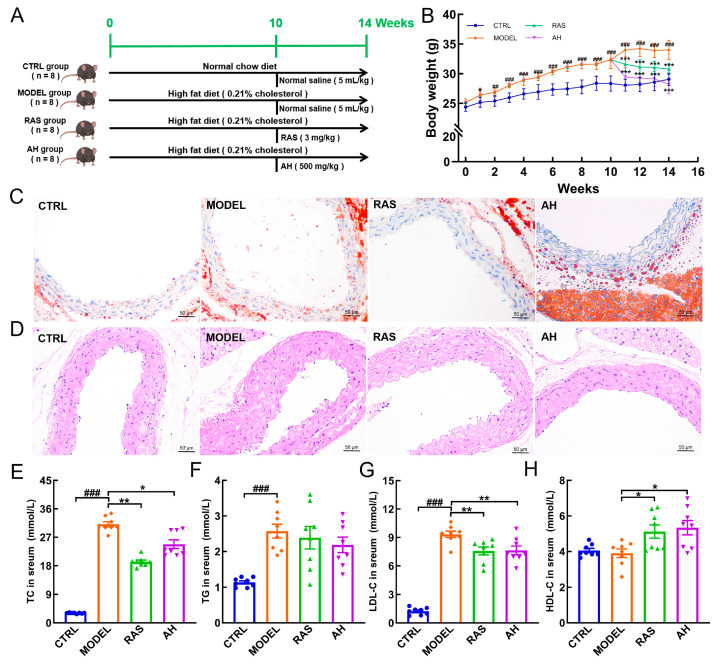
*A. heimuer* alleviated dyslipidemia symptoms in AS mice. (**A**) Developing and treating atherosclerosis models. (**B**) *A. heimuer* administration inhibited body-weight gain in AS mice (n = 8). Histopathological analysis of the aorta was performed using Oil Red O staining (**C**) and H&E staining (**D**) (n = 3) (200×; scale bar: 50 μm). *A. heimuer* administration reduced serum levels of TC (**E**) and LDL-C (**G**) and increased the HDL-C (**H**) levels but did not affect the level of TG (**F**) in AS mice (n = 8). The data are expressed as mean ± S.D. ^#^ *p* < 0.05, ^##^ *p* < 0.01, and ^###^ *p* < 0.001 vs. CTRL mice, * *p* < 0.05, ** *p* < 0.01, and *** *p* < 0.001 vs. MODEL mice.

All animal experiments were approved by the Institutional Animal Ethics Committee of Jilin Agricultural University (Permit No. 20230908002, approval date: 8 September 2023) and conducted in accordance with the ARRIVE guidelines. Eight C57BL/6 mice and twenty-four ApoE^−/−^ mice (male, 8 weeks old, 18–22 g) were purchased from Hangzhou Ziyuan Laboratory Animal Technology Co., Ltd. (License No.: SCXK (Zhe) 2024-0004; Hangzhou, China). Following a one-week acclimatization phase, all C57BL/6 mice were assigned to a normal chow diet to serve as the control (CTRL) group, while all ApoE^−/−^ mice were assigned to an HFD for 14 weeks to establish the AS model, as illustrated in Figure 1A. Owing to its well-established efficacy and widespread clinical application as a first-line therapy for atherosclerosis [22], rosuvastatin (RAS) was employed as the positive control in this study. Beginning from the 11th week, the AS mice were randomly divided into three groups (n = 8) based on body weight: the MODEL group, the RAS group, and the *A. heimuer* (AH) group. During the final four weeks, the CTRL and MODEL groups received 5 mL/kg normal saline (NS) intragastrically (i.g.) daily; the RAS group and the AH group were administered 3 mg/kg RAS (i.g.) and 500 mg/kg AH (i.g.) daily, respectively. Following the final administration, the mice were subjected to a 12 h fasting period prior to blood collection; euthanasia by CO_2_ inhalation; retrieval of cecal contents; and collection of various organs (liver, spleen, kidney, heart, pancreas, and thymus), white adipose tissue (WAT, including epididymal (eWAT), perirenal (pWAT), and inguinal (iWAT) adipose tissue), and scapular brown adipose tissue (BAT).

### 2.2. Organ Index

The indexes of the adipose tissues and organs were calculated as described in a previous study [23]. The organ/adipose tissue index was calculated as follows:

Organ or adipose tissue index (%) = [organ/adipose weight (g)/body weight (g)] × 100%

### 2.3. Biochemical Analysis

Blood samples collected from the above mice were subjected to centrifugation (3000 rpm, 15 min) to obtain serum. Serum lipid concentrations were measured using assay kits according to our previous study [23,24]. Details of assay kits are displayed in Table S1.

### 2.4. Histopathological Analysis

The aforementioned organs, along with adipose tissues and the aorta, were stained by hematoxylin and eosin (H&E) staining and Oil Red O staining as described in our previous study [25]. The detailed protocols are described in the Supplementary Materials.

### 2.5. Immunofluorescence Staining (IF)

Following preprocessing, aorta sections from the CTRL, MODEL, and AH groups (n = 3) were incubated with a dihydroethidium probe in darkness. The sections were then washed three times with phosphate-buffered saline and subsequently counterstained with DAPI for nuclei. Finally, the stained sections were visualized and imaged using a fluorescence microscope (NIKON ECLIPSE CI, Nikon, Tokyo, Japan) [26].

### 2.6. Intestinal Microbiota Analysis

The total microbial genomic DNA was extracted from the cecal contents of the CTRL, MODEL, and AH groups (n = 4) and sequenced using an Illumina MiSeq platform (Illumina, San Diego, CA, USA). The method and data analysis were conducted following the description in our previous study [27,28].

### 2.7. Metabolomics Analysis

Serum samples from the CTRL, MODEL, and AH groups (n = 4) were subjected to non-targeted metabolomic analysis as our previous study [29]. Detailed procedures are provided in the Supplementary Materials.

### 2.8. Western Blot (WB)

The proteins of preprocessed aortic tissue were separated using WB. Detailed procedures are provided in the Supplementary Materials.

### 2.9. Statistical Analysis

Data analysis was conducted using SPSS 16.0 software (IBM Corporation, Armonk, NY, USA). One-way analysis of variance (ANOVA) followed by Dunnett’s *t* test was employed for statistical analysis. For the presentation of statistical results, 95% confidence intervals (95% CIs) were calculated and reported to quantify the precision of estimated effects, providing a range within which the true population parameter is likely to fall with 95% confidence. A *p*-value of less than 0.05 was considered statistically significant in all analyses, and significant differences were further interpreted in conjunction with the corresponding 95% confidence intervals to enhance the robustness of the statistical inferences.

## 3. Results

### 3.1. A. heimuer Relieved Atherosclerotic Symptoms in AS Mice

HFD induces dysregulation of lipid metabolism, exacerbates endothelial injury, and ultimately facilitates the progression of AS [30]. A 14-week HFD significantly increased body-weight gain (*p <* 0.001) (Figure 1B), promoted endothelial lipid accumulation (Figure 1C), and increased vessel wall thickening (Figure 1D) in ApoE^−/−^ mice; these effects were attenuated by *A. heimuer* administration. Compared with the CTRL group, HFD feeding elevated the serum levels of TC (*p* < 0.001) (Figure 1E), TG (*p* < 0.001) (Figure 1F), and LDL-C (*p* < 0.001) (Figure 1G) in AS mice but did not affect HDL-C levels (Figure 1H). Four-week *A. heimuer* administration effectively down-regulated the levels of TC (*p* < 0.05) (Figure 1E) and LDL-C (*p* < 0.01) (Figure 1G) and up-regulated the HDL-C level (*p* < 0.05) (Figure 1H). Interestingly, *A. heimuer* administration did not significantly alter the serum TG level (Figure 1F) in HFD-induced AS mice.

**Figure 2:**
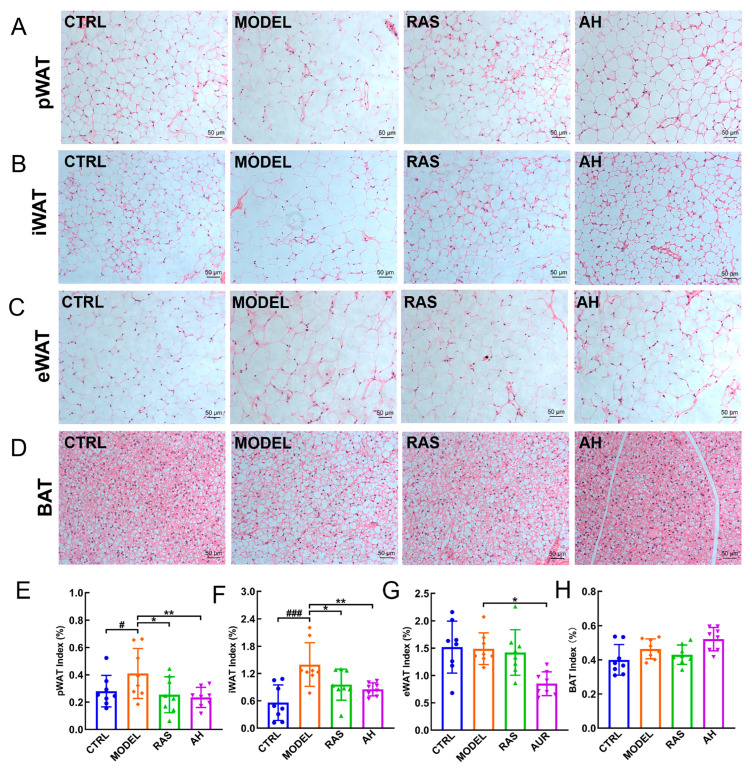
*A. heimuer* reduced fat accumulation in AS mice. H&E staining of pWAT (**A**), iWAT (**B**), eWAT (**C**), and BAT (**D**) (n = 3) (200×; scale bar: 50 μm). *A. heimuer* administration reduced the indexes of pWAT (**E**), iWAT (**F**), and eWAT (**G**) but did not affect the index of BAT (**H**). The data are presented as mean ± S.D. (n = 8). ^#^ *p* < 0.05, and ^###^ *p* < 0.001 vs. CTRL mice; * *p* < 0.05 and ** *p* < 0.01 vs. MODEL mice.

HFD-induced adipocyte hypertrophy was observed in the pWAT (Figure 2A), iWAT (Figure 2B), eWAT (Figure 2C), and BAT (Figure 2D) of ApoE^−/−^ mice. *A. heimuer* treatment significantly reversed these hypertrophies of the above four adipose tissues in AS mice (Figure 2A–D). The HFD also significantly elevated the indexes of pWAT (*p* < 0.05) (Figure 2E) and iWAT (*p* < 0.001) (Figure 2F) while having no significant effect on the indexes of eWAT (Figure 2G) and BAT (Figure 2H). Compared to the MODEL group, four-week *A. heimuer* administration decreased indexes of pWAT (*p* < 0.01) (Figure 2E), iWAT (*p* < 0.01) (Figure 2F), and eWAT (*p* < 0.05) (Figure 2G) and did not affect the BAT (Figure 2H) index in AS mice.

Additionally, the pathological effects of *A. heimuer* on major organs and its impact on organ indices were further investigated. The histopathological findings indicated that *A. heimuer* inhibited the accumulation of lipid vacuoles in the liver while exerting no influence on the kidney, heart, spleen, or thymus in HFD-induced ApoE^−/−^ mice. Furthermore, *A. heimuer* treatment reduced the indexes of the heart, liver, spleen, and thymus (*p* < 0.05) (Table S3), without affecting the kidney or pancreas in HFD-induced ApoE^−/−^ mice (Table S3).

### 3.2. A. heimuer Regulated the Intestinal Microflora in AS Mice

**Figure 3:**
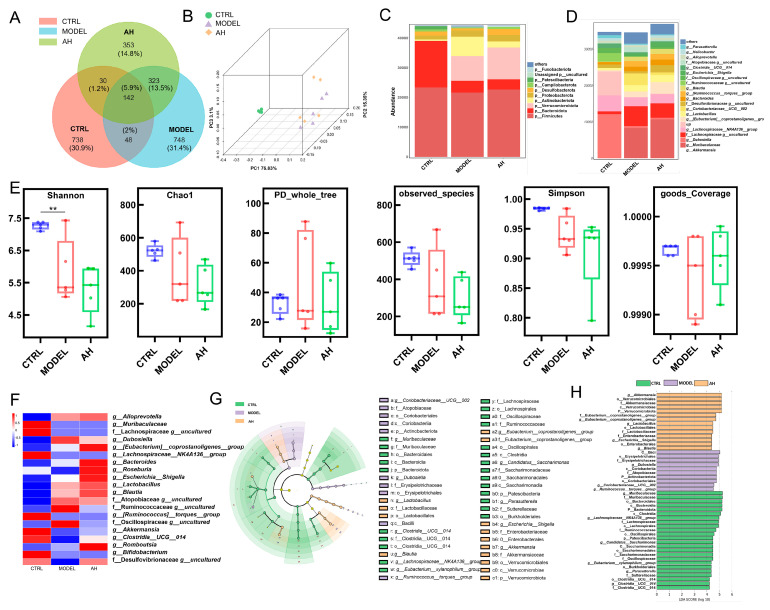
*A. heimuer* altered the intestinal microbiota in AS mice. (**A**) Venn diagram of the three groups. (**B**) Beta diversity. (**C**) Histogram of the relative abundance of species at the phylum level. (**D**) Histogram of the relative abundance of the top 20 at the genus level. (**E**) Alpha diversity. ** *p* < 0.01 vs. CTRL mice. (**F**) Heatmap of the top 20 differential genera. (**G**,**H**) Taxonomic lineage map based on LEfSe analysis (n = 4).

Alterations in the intestinal microbiota are known to significantly influence the progression of AS [31]. A Venn diagram analysis of the 2382 detected operational taxonomic units (OTUs) revealed that 142 (5.9%) were common OTUs to the CTRL, MODEL, and AH groups (Figure 3A). There were also 738 (30.9%), 748 (31.4%), and 353 (14.8%) unique OTUs in the CTRL, MODEL, and AH groups, respectively (Figure 3A). Beta and alpha diversity analysis demonstrated that *A. heimuer* administration altered the composition and distribution of microflorae in AS mice (Figure 3B,E). At the phylum level, an increase in the abundance of Actinobacteriota, Proteobacteria, etc., and a reduction in the abundance of Firmicutes, Desulfobacterota, and Campilobacterota were noted in AS mice compared with CTRL mice, which were reversed by *A. heimuer* administration (Figure 3C). Moreover, at the top-20 genus level, long-term HFD elevated the abundance of *g_Ruminococcus_torques_group* and decreased the abundance of *Akkermansia* in comparison with the CTRL group (Figure 3D,F). Four-week *A. heimuer* administration altered these abundances in AS mice (Figure 3D,F). LEfSe analysis showed that the *Lactobacillus*, *Akkermansia*, *Eubacterium_coprostanoligenes_group*, *Escherichia_Shigella*, and *Blautia* genera were significantly enriched in the gut microbiota of AS mice following *A. heimuer* treatment (Figure 3G).

### 3.3. A. heimuer Altered Serum Metabolism in AS Mice

**Figure 4:**
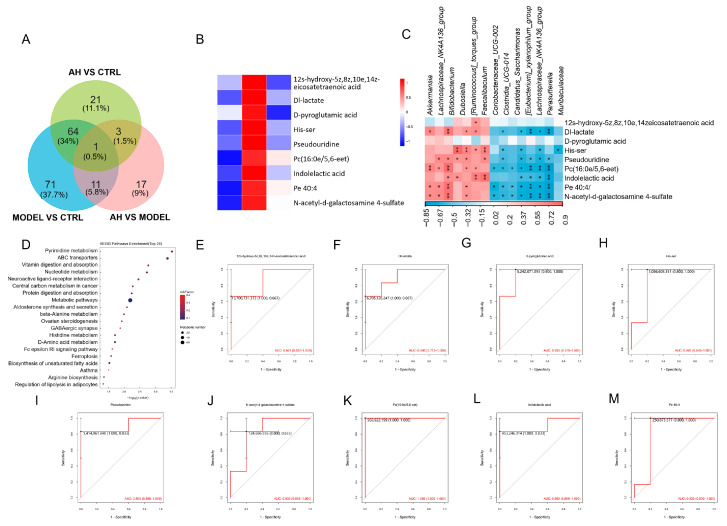
*A. heimuer* modulated serum metabolite contents in AS mice. (**A**) Venn diagram. (**B**) Heatmap of significantly altered metabolites among the three groups. (**C**) Correlation heatmap between significantly altered metabolites and differential gut microbiota at the top-15 level. * *p* < 0.05, and ** *p* < 0.01 (**D**) The KEGG enrichment pathway. (**E**–**M**) The area under the ROC curve (AUC) values of significantly altered metabolites.

To further elucidate the potential mechanisms by which *A. heimuer* alleviates AS, serum samples from the CTRL, MODEL, and AH groups were analyzed using non-targeted metabolomics. A total of 188 differential metabolites were identified utilizing untargeted metabolomics analysis (Figure 4A). Among them, 89 differential metabolites were obtained in the AH vs. CTRL group, 32 differential metabolites were obtained in the AH vs. MODEL group, and 147 differential metabolites were obtained in the MODEL vs. CTRL group (Figure 4A). Compared with the CTRL group, HFD significantly up-regulated the levels of 12(S)-hydroxy-5Z,8Z,10E,14Z-eicosatetraenoic acid (12(S)-HETE), Dl-lactate, D-pyroglutamic acid, His-ser, Pseudouridine, Pc(16:0e/5,6-eet), Indolelactic acid, Pe 40:4, and N-acetyl-d-galactosamine 4-sulfate. Treatment with *A. heimuer* effectively reversed these HFD-induced metabolic alterations. Kyoto Encyclopedia of Genes and Genomes (KEGG) pathway enrichment analysis showed that these significantly altered metabolites might be involved in the regulation of lipolysis in adipocytes and the biosynthesis of adipocytes’ unsaturated fatty acids (Figure 4D). To evaluate the diagnostic potential of these metabolites for atherosclerosis, we conducted Receiver Operating Characteristic (ROC) analysis. The results revealed that the aforementioned metabolites yielded area under the curve (AUC) values greater than 0.7, indicating a strong correlation with AS (Figure 4E–M). Notably, 12(S)-HETE, an endogenous ligand for LTB4R2, is known to be associated with inflammation [32]. Furthermore, a correlation analysis between metabolites and microbiota revealed that 12(S)-HETE was positively correlated with *g_Ruminococcus_torques_group*, while N-acetyldgalactosamine 4-sulfat was positively correlated with *Akkermansia*. This suggests that the underlying mechanisms by which *A. heimuer* alleviates AS may involve the modulation of inflammation (Figure 4C).

### 3.4. A. heimuer Modulated the Nrf2/NF-κB Signaling Pathway in AS Mice

**Figure 5:**
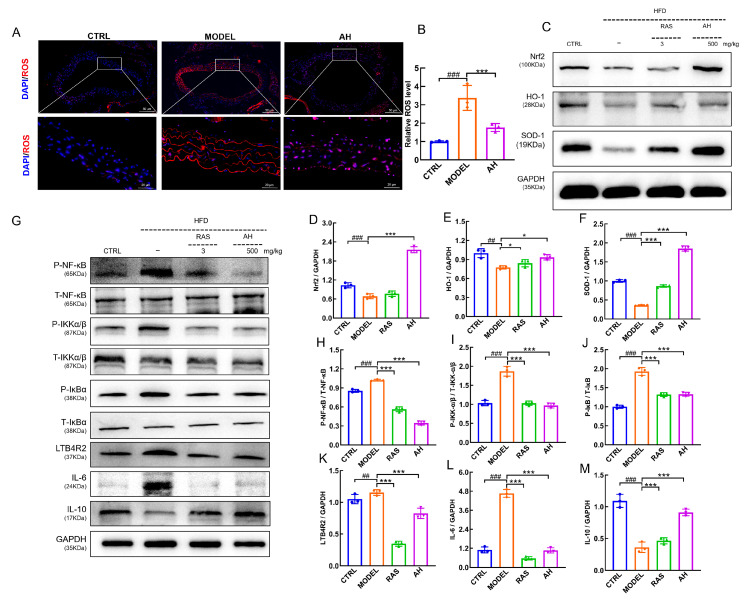
*A. heimuer* modulated the Nrf2/NF-κB signaling pathway in AS mice. (**A**) *A. heimuer* reduced ROS levels in the aorta. (**B**) Semi-quantitative analysis of ROS in the aorta. (**C**) Western blot analysis of Nrf2, HO-1, and SOD1. (**D**–**F**) Quantifications of protein expression of Nrf2, HO-1, and SOD1. (**G**) Western blot analysis of P-NF-κB, P-IKKα/β, P-IκBα, LTB4R2, IL-6, and IL-10. (**H**–**M**) Quantifications of protein expression of P-NF-κB, P-IKKα/β, P- IκBα, LTB4R2, IL-6, and IL-10. Data are expressed as mean ± S.D. (n = 3). ^##^ *p* < 0.01, and ^###^ *p* < 0.001 vs. the CTRL group; * *p* < 0.05, and *** *p* < 0.001 vs. MODEL group.

Oxidative stress and inflammation synergistically contribute to the progression of AS [33]. Vascular endothelial ROS levels were significantly elevated in AS mice compared to the CTRL mice (*p* < 0.05) (Figure 5A,B), and this elevation was notably reduced following *A. heimuer* administration (*p* < 0.05) (Figure 5A). Activation of Nrf2 up-regulates the expression of antioxidant enzymes and inhibits ROS generation, thereby alleviating oxidative stress injury and atherosclerosis in endothelial cells [34]. Consistent with this, *A. heimuer* administration strongly enhanced the expression of Nrf2 (*p* < 0.05) (Figure 5C,D); its downstream effector, HO-1 (*p* < 0.05) (Figure 5C,E); and SOD-1 (*p* < 0.05) (Figure 5C,F) in the aorta of HFD-induced AS mice. Dysregulation of lipid metabolism can trigger the onset of an inflammatory response, resulting in the up-regulation of pro-inflammatory factor expression and the activation of the NF-κB signaling pathway [35]. Four-week *A. heimuer* administration effectively decreased the expression levels of P-NF-κB (*p* < 0.05) (Figure 5G,H), P-IKKα/β (*p* < 0.05) (Figure 5G,I), P-IκBα (*p* < 0.05) (Figure 5G,J), LTB4R2 (*p* < 0.05) (Figure 5G,K), and IL-6 (*p* < 0.05) (Figure 5G,L) and markedly increased the expression level of IL-10 (*p* < 0.05) (Figure 5G,M). Overall, these results suggest that *A. heimuer* administration modulated the Nrf2/NF-κB signaling pathway to relieve atherosclerotic symptoms in AS mice.

## 4. Discussion

In the pathogenesis of AS, LDL is an important indicator for dyslipidemia [36]. Dyslipidemia can induce oxidative stress and inflammatory responses through multiple mechanisms [37]. In this study, *A. heimuer* significantly reduced both aortic wall thickness and serum LDL-C levels in HFD-induced ApoE^−/−^ mice, which is consistent with previous studies [16]. Furthermore, *A. heimuer* mitigated HFD-induced adipocyte hypertrophy in pWAT, iWAT, eWAT, and BAT, thereby inhibiting fat accumulation. Taken together, these results suggest that *A. heimuer* restored lipid metabolism to suppress atherosclerotic lesions in AS mice.

Alterations in the intestinal microbiota influence the progression of AS [38]. *Akkermansia* is negatively correlated with obesity, diabetes, and cardiovascular metabolic diseases [39]. Supplementation with *Akkermansia* has been shown to reverse HFD-induced metabolic disorders and insulin resistance in obese mice [40]. Furthermore, *Akkermansia* may exert anti-atherosclerotic effects by reducing blood levels of lipopolysaccharides and alleviating metabolic endotoxemia [40]. *Ruminococcus* acts as a pro-inflammatory marker, with its abundance positively correlated with intestinal disorders like Crohn’s disease [41] and strongly related to obesity [42]. Notably, its abundance increases significantly in obese individuals and decreases following weight loss, which is similar to the changes observed in obese dogs and rats [42]. In this study, we found that *A. heimuer* treatment modulated the structure and composition of the intestinal microbiota in HFD-induced ApoE^−/−^ mice, leading to an increased abundance of the *Akkermansia* genus while concurrently decreasing the abundance of *[Ruminococcus]_torques_group*. These findings suggest that *A. heimuer* treatment improves microbiome composition in AS mice.

The intestinal microbiota is known to modulate the cardiovascular system via its metabolites [43]. In this study, a correlation analysis of the microbiota and metabolites revealed a significant positive relationship between *[Ruminococcus]_torques_group* and 12(S)-HETE, as well as between *Akkermansia* and N-acetyl-d-galactosamine 4-sulfate. 12(S)-HETE, an endogenous ligand for LTB4R2, is associated with oxidative stress. Furthermore, LTB4R2 can bind to NF-κB, activating a pro-inflammatory response [44]. In contrast, N-acetyl-d-galactosamine 4-sulfate may reduce inflammation by modulating intestinal homeostasis [45]. Our results demonstrate that *A. heimuer* reduced the contents of 12(S)-HETE and the expression of LTB4R2 while concurrently enhancing the levels of N-acetyl galactosamine 4-sulfate in HFD-induced ApoE^−/−^ mice. These findings indicate that *A. heimuer* may contribute to the mitigation of AS by collaboratively regulating both the intestinal microbiota and metabolites.

Oxidative stress facilitates the progression of AS by enhancing LDL peroxidation, leading to vascular endothelial damage and stimulating the release of inflammatory mediators [46]. As a powerful oxidizing agent, ROS are naturally produced during cellular metabolism [47], but their excessive production can induce oxidative stress, damaging the vascular wall and facilitating inflammatory cell infiltration [46]. In response, the activation of Nrf2 promotes the expression of downstream antioxidant enzymes such as HO-1 and SOD-1, which are responsible for removing ROS and lipid peroxide products [48]. Conversely, inflammation is largely driven by the NF-κB pathway. Pro-inflammatory cytokines like IL-6 activate the IKK complex, leading to the phosphorylation of inhibitor protein IκBα [49]. This process releases NF-κB, allowing it to translocate to the nucleus and initiate the transcription of target genes [50]. However, IL-10 is a critical anti-inflammatory cytokine that acts as a master regulator to suppress NF-κB-driven inflammation [51]. In this study, *A. heimuer* treatment enhanced the expressions of Nrf2, HO-1, SOD-1, and IL-10 while decreasing the expression of IL-6 and the phosphorylation levels of NF-κB, IKKα/β, and IκBα in AS mice. These results suggest that *A. heimuer* regulated oxidative stress and inflammation to ameliorate atherosclerosis via the Nrf2/NF-κB signaling pathway.

There are some limitations of this study. First, while the efficacy of *A. heimuer* is evident, the specific bioactive components responsible for its anti-atherosclerotic effects remain to be elucidated. Secondly, the relationship between the therapeutic effect and dosage is unclear, and a dose-dependent response must be confirmed. Finally, although our findings suggest that the improvement in AS is linked to the regulation of intestinal flora and serum metabolites, the detailed molecular mechanisms involved require further in-depth investigation.

## 5. Conclusions

In this study, *A. heimuer* significantly inhibited body-weight gain, regulated lipid levels, and alleviated aortic lesions in HFD-induced ApoE^−/−^ mice. Through integrated analysis of the intestinal microbiota and serum metabolomics, we found that *A. heimuer* exerted its therapeutic effects by modulating elements of the gut microbiome, including *Akkermansia* and *Ruminococcus*, and elements of the serum metabolome, such as 12(S)-HETE and N-acetyl-d-galactosamine 4-sulfate. Furthermore, *A. heimuer* alleviated oxidative stress and inflammatory responses to mitigate atherosclerosis via the Nrf2/NF-κB signaling pathway in AS mice. These results provide a theoretical foundation and empirical evidence supporting *A. heimuer* as a potential therapeutic strategy for the treatment of atherosclerosis.

## Data Availability

The original contributions presented in this study are included in the article/Supplementary Material. Further inquiries can be directed to the corresponding author.

## Supplementary Materials

The supporting information is available for download at: https://www.mdpi.com/article/10.3390/nu17172799/s1, Supplementary methods. Figure S1. H&E staining of heart (A), liver (B), spleen (C), kidney (D), thymus (E), and pancreas (F) (100×; scale bar: 100 μm); Table S1. Details of commercially available test kits. Table S2. Details of antibodies used in Western blot; Table S3. The organ index in ApoE^−/−^ mice. The indexes of the heart (A), liver (B), spleen (C), kidney (D), thymus (E), and pancreas. All data are expressed as mean ± S.E.M. ^##^ *p* < 0.01, ^###^ *p* < 0.001 vs. CTRL mice. * *p <* 0.05, ** *p <* 0.01, *** *p <* 0.001 vs. MODEL mice; Table S4. Abundance of top 10 phyla among CTRL, MODEL, and AH groups. Data are presented as the mean; Table S5. Relative abundance (log10) of the top 20 genera among CTRL, MODEL, and AH groups. Data are presented as the mean; Table S6. The differential metabolites of serum among CTRL, MODEL, and AH groups. Data are presented as the mean. Differences were considered statistically significant at *p <* 0.05 and VIP > 1.
