# Supplementary material for: Auricularia heimuer Ameliorates Oxidative Stress and Inflammation to Inhibit Atherosclerosis Development in ApoE−/− Mice
Source: Nutrients. 2025 Aug 28;17(17):2799. doi: 10.3390/nu17172799 (PMC12430062; doi:10.3390/nu17172799)
Supplement: Supplementary file 1 [file nutrients-17-02799-s001.zip › nutrients-3764972-supplementary.pdf]

Auricularia heimuer ameliorates oxidative stress and inflammation to inhibit atherosclerosis development in ApoE$^{-/-}$ mice.

Jundi Zhao [1,4], Siyu Ma [1,4], Yifan Hu [1,4], Jing Ling [1,4], Zhuqian Wang [5], Jingyu Wang [2*], Junliang Chen[1,3*], and Yongfeng Zhang [1,4*]

[1]Engineering Research Center of Chinese Ministry of Education for Edible and Medicinal Fungi, 130118, China;
[2]Jilin Academy of Agricultural Sciences; Changchun 130033, China
[3]Science and Research Center for Edible Fungi of Qingyuan County, Qingyuan 323800, China
[4]College of Plant Protection, Jilin Agricultural University, Changchun 130118, China;
[5]Clinical Research Center for Advanced Diagnostics and Therapeutics, Zhongshan People's Hospital, Zhongshan 528403, Guangdong, China;
[*]Correspondence: jlskjtwjy@163.com(J.W.); chenjunliang1026@126.com (J.C.); zhang-yongfeng@jlau.edu.cn (Y.Z.

Jingyu Wang, Professor, Ph.D
Jilin Academy of Agricultural Sciences,
Changchun, Jilin, P.R.China.
Email: jlskjtwjy@163.com

Junliang Chen, Ph.D
Engineering Research Center of Chinese Ministry of Education for Edible and Medicinal Fungi, Jilin Agricultural University,
Changchun, Jilin, P.R.China.
Science and Research Center for Edible Fungi of Qingyuan County,
Qingyuan, Zhejiang, P.R.China.
Email: chenjunliang1026@126.com

Yongfeng Zhang, Ph.D

Engineering Research Center of Chinese Ministry of Education for Edible and Medicinal Fungi, Jilin Agricultural University,
Changchun, Jilin, P.R.China.
College of Plant Protection, Jilin Agricultural University,
Changchun, Jilin, P.R.China.
Email: zhangyongfeng@jlau.edu.cn

Materials and Methods

1. Animal treatment and Randomization

Eight male C57BL/6J and 24 male ApoE-/- mice (8 weeks old, 18-22 g, of specific pathogen-free grade) were purchased from Hangzhou Ziyuan Laboratory Animal Technology Co., Ltd. (license No.: SCXK (zhe) 2024-0004; Zhejiang, China). All mice were maintained under the following conditions: temperature, 23 ± 1°C; humidity, 50 ± 10%; and a 12-h light/dark cycle (7 am to 7 pm). After 7 days of acclimatization, ApoE-/- mice were each fed high-fat diet (HFD, 21% cholesterol), whereas eight C57BL/6J mice were each fed normal chow for the same period. The mice were provided with food and water ad libitum throughout the experiment. Following the completion of the modeling process, the twenty-four mice in the model group were assigned unique identification numbers. The numbers 1 through 24 (representing the mouse IDs) were entered in cells A1 to A24 in Excel. In cell B1, the random number function =RAND () was input, and the fill handle was dragged down to cell B24 to generate a random number for each mouse. Select the data range A1:B24, navigate to the "Data" tab, click "Sort," and sort the dataset based on the values in column B. After sorting, allocate the first four mice to Cage 1, the next four to Cage 2, and continue in this manner until all six cages have been assigned. Following the numbering of the cages, repeat the aforementioned procedure. Randomly allocate the six cages of mice into three groups: the MODEL group, the rosuvastatin (RAS) group, and the *Auricularia heimuer* (AH) group.

2. Blinding

During the sample preparation stage, the research team uniformly encoded all experimental animal samples (including intestinal flora samples and serum metabolite samples). Each group of samples was assigned a unique random code, and these codes were completely independent of the original group information (e.g., control group, experimental group). Only an independent member of the research team, who was responsible for data management, retained access to the code comparison table and did not participate in any subsequent testing procedures. When the samples were submitted to the testing institution, only the coded samples and the specific testing requirements (e.g., "16S rRNA sequencing of intestinal flora", "untargeted metabolomics analysis of serum") were provided, without disclosing any group identities, treatment protocols, or experimental objectives to the testing personnel. Throughout the entire testing and raw

data analysis process, the testing personnel recorded results solely based on the assigned random codes and remained unaware of the sample groupings. Once all testing data were collected, the research team used the code comparison table to match and integrate the data by group. This procedure ensured that all experimental operations were conducted in a blinded manner, effectively minimizing the potential influence of subjective bias on the testing outcomes.

3. Histopathological analysis

For H&E staining, paraffin sections were first deparaffinized by immersion in xylene I and II for 10 minutes each. They were then rehydrated through a graded ethanol series (100%, 95%, 80%, 70%) for 5 minutes per step and rinsed with distilled water for 2 minutes. Next, the sections are stained with hematoxylin solution for 5 minutes, rinsed with tap water to remove excess dye, differentiated using 1% hydrochloric acid in ethanol for a few seconds, and blued under running tap water for 5 minutes. Following this, the sections are rinsed with distilled water for 1 minute, counterstained with eosin solution for 2 minutes, and lightly rinsed again with tap water. Finally, the sections are dehydrated in 95% ethanol I and II (2 minutes each), followed by 100% ethanol I and II (2 minutes each), cleared in xylene I and II (5 minutes each), and mounted with neutral gum. For Oil Red O staining, if using a frozen section, it should be equilibrated at room temperature for 30 minutes and subsequently rinsed with distilled water for 2 minutes. In the case of a paraffin section, dewaxing to water is required prior to staining. Following this preparation, the section is immersed in 60% isopropanol for 5 minutes, air-dried, and then placed in preheated Oil Red O staining solution at 37°C for 10 minutes. The section is then differentiated in 60% isopropanol until the background becomes colorless, which typically takes 1 minutes, followed by a rinse with distilled water. Next, the section is counterstained with hematoxylin solution for 3 minutes, rinsed with tap water, and differentiated using 1% hydrochloric acid in ethanol for nuclear bluing. Finally, the section was mounted with glycerol gelatin; it is crucial to avoid xylene-based clearing agents, as Oil Red O is a lipid-soluble dye and may be extracted during this step.

4. Metabolomics analysis

Serum samples were analyzed using ul-tra-high-performance liquid chromatography (UHPLC, model 1290 Infinity LC; Ag-ilent Technologies, Santa Clara, CA, USA) along with triple quadrupole mass spec-trometry (AB SCIEX TripleTOF 6600; AB SCIEX, Framingham, MA, USA). Correlations among significantly different

metabolites were exam-ined through correlation analysis methods ($P < 0.05$). The relevant metabolic pathways were analyzed by utilizing the Kyoto Encyclopedia of Genes and Genomes (KEGG). Receiver Operating Characteristic (ROC) analysis can be used to assess metabo-lite-disease correlations. An AUC value greater than 0.7 indicates that the metabolite can be used as a marker for disease diagnosis, and the closer the AUC value is to 1, the higher the reference value of the metabolite as a disease marker.

5. Western blot (WB)

Aortic tissue was homogenized and lysed in ice-cold RIPA buffer containing protease and phosphatase inhibitors. Protein samples concentrations were measured using the Pierce™ BCA Protein Assay Kit. Thirty micrograms of protein was separated via 12% sodium dodecyl sulfate-polyacrylamide gel electrophoresis, followed by transfer onto a polyvinylidene fluoride membrane. After being blocked with rapid blocking buffer for 15 min, the membranes were incubated with the primary antibodies overnight at 4°C and then with secondary antibodies at 4°C for 4 h. Subsequently, an ECL Chemiluminescent Substrate Detection Kit was utilized for detection. Densitometric analysis of the pro-tein bands was performed using ImageJ software (version 6.0; National Institutes of Health, Bethesda, MD, USA). Details of the reagents and antibodies used were provid-ed in Table S2.

Supplementary Figures

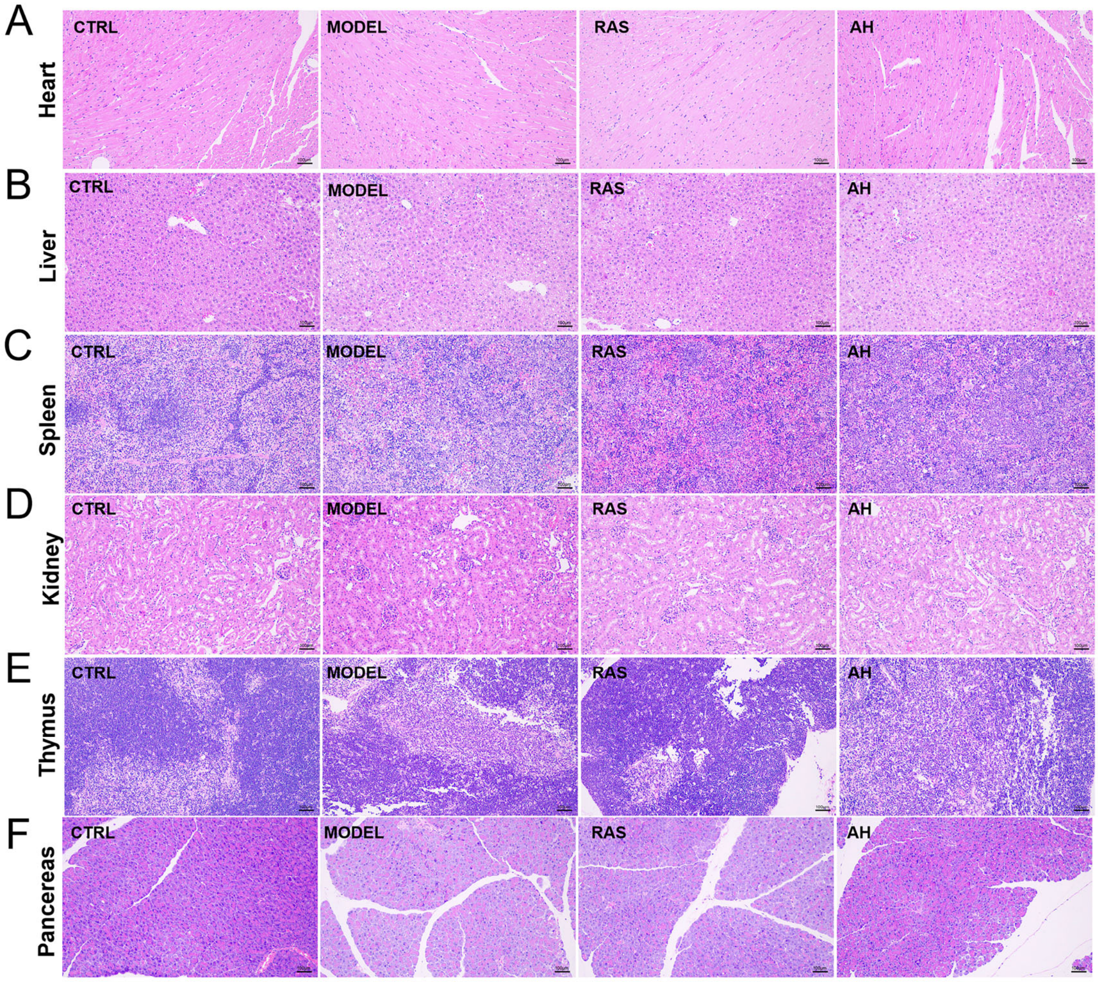

Figure S1. H&E staining of heart (A), liver (B), spleen (C), kidney (D), thymus (E), and pancereas (F) (100×; scale bar: 100 μm).

Supplementary Tables

Table S1. Details of commercially available test kits.

Cytokines	Catalog number	Company	Area
TC	A111-1-1	Nanjing Jiancheng Biotechnology Research Institute	Jiangsu, China
TG	A110-1-1	Nanjing Jiancheng Biotechnology Research Institute	Jiangsu, China
LDL-C	A113-1-1	Nanjing Jiancheng Biotechnology Research Institute	Jiangsu, China
HDL-C	A112-1-1	Nanjing Jiancheng Biotechnology Research Institute	Jiangsu, China

Table S2 Detail information of antibodies used in western blot.

Antibody	Molecular weight	Catalog number	Dilution
Anti-Nrf2 [a]	100 kDa	a1244	1:1000
Anti-SOD1 [a]	16 kDa	A12537	1:1000
Anti-HO-1 [b]	33 kDa	AF5393	1:1000
Anti- NF-κB p65 (phosphor Ser536) [b]	65 kDa	AF2006	1:1000
Anti-NF-κB [b]	60 kDa	ab32536	1:1000
Anti-LTB4R2 [b]	38 kDa	DF15894	1:1000
Anti-IKKα/β [b]	85 kDa,87 kDa	AF6041	1:1000
Anti-IKKα/β (phosphor Ser176/180) [c]	85-87 kDa	2697s	1:1000
Anti-IKB[b]	39 kDa	AF7776	1:1000
Anti-IKB (phosphor Ser32) [c]	40 kDa	2859s	1:1000
Anti-IL-6 [b]	23 kDa	bs-0782R	1:1000
Anti-IL-10 [b]	19 kDa	DF6894	1:1000
Anti-GAPDH [d]	37 kDa	E-AB-48016	1:4000
goat anti- rabbit [d]		E-AB-1003	1:4000

[a] Wuhan ABclonal Technology Co., Ltd., Wuhan, Hubei, China; [b] Affinity Biosciences, Cincinnati, OH, USA; [c] Cell Signaling Technology Co., Ltd, Massachusetts, USA; [d] Elabscience Biotechnology Co., Ltd., Wuhan, China.

Table S3 The organ index in $ApoE^{-/-}$ mice

	Organ	CTRL	HFD		
			MODEL	RAS	AH
Organ Index (%)	Heart	0.64±0.03	0.76±0.03#	0.64±0.02*	0.57±0.14**
	Liver	4.71±0.18	5.82±0.21##	5.12±0.08*	4.15±0.62***
	Spleen	0.27±0.01	0.93±0.09###	0.58±0.06**	0.71±0.04*
	Kidney	1.26±0.01	1.43±0.04	1.39±0.05	1.40±0.05
	Thymus	0.127±0.01	0.123±0.01	0.111±0.01	0.091±0.01*
	Pancreas	0.84±0.01	0.74±0.07	0.68±0.1	0.77±0.11

Data are presented as the mean

Table S4 Abundance of top 10 phylum among CTRL, MODEL, and AH groups.

Taxa	CTRL	MODEL	AH
p__Firmicutes	138501	132508	113161
p__Bacteroidota	93891	20765	17199
p__Verrucomicrobiota	1690	56480	53409
p__Actinobacteriota	2706	35127	10427
p__Proteobacteria	8134	10772	10663
p__Desulfobacterota	8737	4269	10209
p__Campilobacterota	3947	1226	3498
p__Patescibacteria	6392	485	61
Unassigned|p__uncultured	95	1556	1015
p__Deferribacterota	68	419	623

Data are presented as the mean

Table S5. Relative abundance (log10) of the top 20 genus among CTRL, MODEL, and AH groups.

Taxa	CTRL	MODEL	AH
g__Alloprevotella	0.007182458	0.211925505	0.24162386
g__Muribaculaceae	0.27593854	0.011807834	0.009766695
f__Lachnospiraceae g__uncultured	0.10961508	0.057921296	0.057364622
g__Dubosiella	0.016111878	0.12770021	0.089859926
g__[Eubacterium]_coprostanoligenes_group	0.004077753	0.03246815	0.07196488
g__Lachnospiraceae_NK4A136_group	0.139603087	0.00316354	0.003032292
g__Bacteroides	0.017487724	0.024511778	0.03443235
g__Roseburia	0.025480301	0.009300536	0.04579213
g__Escherichia-Shigella	0.00033491	0.014401122	0.029182413
g__Lactobacillus	0.004322147	0.03577199	0.04403159
g__Blautia	0.003131859	0.023321491	0.030893168
f__Atopobiaceae g__uncultured	0	0.039383585	0.03490756
f__Ruminococcaceae g__uncultured	0.000153877	0.057065918	0.015595936
g__[Ruminococcus]_torques_group	0.056984454	0.001276278	0.001307959
f__Oscillospiraceae g__uncultured	0.003389831	0.032590346	0.007933742
g__Akkermansia	0.033196805	0.003186169	0.010223801
g__Clostridia_UCG-014	0.015469213	0.010092553	0.013165577
g__Romboutsia	0	0.004091331	0.019687267
g__Bifidobacterium	0.02328981	0.00850852	0.007992578
f__Desulfovibrionaceae g__uncultured	0.01612093	0.002271956	0.015804123
g__Alloprevotella	0.007182458	0.211925505	0.24162386

Data are presented as the mean.

Table S6. The differential metabolites of serum among CTRL, MODEL, and AH groups.

Group	CTRL	MODEL	AH	CTGRL *vs* MODEL		MODEL *vs* AH	
				VIP	*P*-value	VIP	*P*-value
Pseudouridine	491928.3485	1864488.425	897524.9695	2.447	0.000	1.987	0.035
Pe 40:4	6043.505224	307595.5428	150772.8766	1.983	0.000	1.045	0.032
Pc(16:0e/5,6-eet)	3941.128834	610812.446	285132.2757	2.292	0.000	1.056	0.001
N-acetyl-d-galactosamine 4-sulfate	13919.93651	177631.1027	79465.77277	1.261	0.002	1.025	0.004
Indolelactic acid	126612.4743	567364.2906	278119.6407	1.564	0.001	1.124	0.013
His-ser	542867.3808	1354638.805	890055.915	2.831	0.000	4.507	0.002
Dl-lactate	44659.53391	7005973.761	3804307.265	5.495	0.000	5.577	0.046
12s-hydroxy-5z,8z,10e,14z-eicosatetraenoic acid	8496532.625	15066831.88	10955243.32	7.828	0.027	3.866	0.029
D-pyroglutamic acid	1629451.942	6844679.687	3437327.208	1.912	0.003	2.081	0.010

Data are presented as the mean. Differences were considered statistically significant at $P < 0.05$ and VIP > 1.
